# Digital home care interventions and quality of primary care for older adults: a scoping review

**DOI:** 10.1186/s12877-024-05120-z

**Published:** 2024-06-10

**Authors:** Ísis de Siqueira Silva, Aguinaldo José de Araújo, Rayssa Horácio Lopes, Cícera Renata Diniz Vieira Silva, Pedro Bezerra Xavier, Renan Cabral de Figueirêdo, Ewerton William Gomes Brito, Luís Velez Lapão, Cláudia Santos Martiniano, Vilani Medeiros de Araújo Nunes, Severina Alice da Costa Uchôa

**Affiliations:** 1 Postgraduate in Collective Health, Federal University of Rio Grande Do Norte, Natal, Brazil; 2 Health School, Federal University of Rio Grande Do Norte, Natal, Brazil; 3 Technical School of Health of Cajazeiras, Teacher Training Center, Universidade Federal of Campina Grande, Cajazeiras, Brazil; 4 Postgraduate in Health Sciences, Federal University of Rio Grande Do Norte, Natal, Brazil; 5 Postgraduate in Family Health, Federal University of Rio Grande Do Norte, Natal, Brazil; 6 Public Health Department, Federal University of Rio Grande Do Norte, Natal, Brazil; 7 WHO Collaborating Center on Health Workforce Policy and Planning, IHMT, Universidade Nova de Lisboa, Lisbon, Portugal; 8 UNIDEMI, Department of Mechanical and Industrial Engineering, Nova School of Science and Technology, Caparica, Portugal; 9 Laboratório Associado de Sistemas Inteligentes, Escola de Engenharia Universidade do Minho, Campus Azurém, 4800-058 Guimarães, Portugal; 10 Department of Nursing, State University of Paraíba, Campina Grande, Brazil

**Keywords:** Scoping review, Digital health, Primary health care, Home-based primary care, Geriatric care, Quality in healthcare

## Abstract

**Background:**

Population aging is forcing the transformation of health care. Long-term care in the home is complex and involves complex communication with primary care services. In this scenario, the expansion of digital health has the potential to improve access to home-based primary care; however, the use of technologies can increase inequalities in access to health for an important part of the population. The aim of this study was to identify and map the uses and types of digital health interventions and their impacts on the quality of home-based primary care for older adults.

**Methods:**

This is a broad and systematized scoping review with rigorous synthesis of knowledge directed by the guidelines of the Preferred Reporting Items for Systematic Reviews and Meta-Analyses extension for Scoping Reviews (PRISMA-ScR). The quantitative data were analyzed through descriptive statistics, and the qualitative data were analyzed through basic qualitative content analysis, considering the organizational, relational, interpersonal and technical dimensions of care. The preliminary results were subjected to consultation with stakeholders to identify strengths and limitations, as well as potential forms of socialization.

**Results:**

The mapping showed the distribution of publications in 18 countries and in the Sub-Saharan Africa region. Older adults have benefited from the use of different digital health strategies; however, this review also addresses limitations and challenges, such as the need for digital literacy and technological infrastructure. In addition to the impacts of technologies on the quality of health care.

**Conclusions:**

The review gathered priority themes for the equitable implementation of digital health, such as access to home caregivers and digital tools, importance of digital literacy and involvement of patients and their caregivers in health decisions and design of technologies, which must be prioritized to overcome limitations and challenges, focusing on improving quality of life, shorter hospitalization time and autonomy of older adults.

**Supplementary Information:**

The online version contains supplementary material available at 10.1186/s12877-024-05120-z.

## Introduction

Home Based Primary Care (HBPC), offered by Primary Health Care (PHC) teams, has been highlighted in patients’ residences in the face of the need for long-term care offered to older adults [1]. However, the continuity of health care in the home environment represents a challenge for PHC professionals, as it requires their integration through efficient communication between home services and those provided at the health center. In this way, to overcome this distance, digital health has been adopted as a solution [2].

Digital health interventions can contribute to strengthening health systems by quickly making reliable and upgraded information available [3]. Studies show that digital health in the home improves access to health care, increases the sense of security and reduces displacement [2, 4–6].

The use of digital health services (e.g., *mHealth*) has been presented as a solution for improving the quality and coverage of services, especially in long-term care follow-up [7]. Various technologies (sensors, apps, chatbots with artificial intelligence, among others) can be used in the prevention of falls in older people [8, 9] increase the independence and well-being of people with dementia [10] and improve the physical and mental health status of patients [11].

However, although the use of these technologies can strengthen health systems and provide support to patients at home, it can also widen inequalities in access to health care. This is because low-and middle-income countries face financial, geographical and human resource obstacles in implementing these technologies, as well as challenges related to governance, infrastructure, literacy and democratization of access [12].

In addition to the challenges associated with the implementation of digital health, in underdeveloped and developing countries, health systems also have to address the barriers of accessing health care for older adults, weak or unstructured public health, lack of gerontological knowledge and ageism [13, 14]. Ageism can affect the use, adoption, and design of technological products and services [15]. The design decisions of digital health strategies often include generalizations that portray older adults as vulnerable and as having lower technological capabilities [16]. The implementation of technologies in a disorderly manner can generate ethical problems related to the data security, privacy and individuality of the subjects [10].

Emphasizing the problems that public health was already facing, the COVID-19 pandemic put further pressure on health systems around the world (e.g., many seniors were left without health care) and accelerated the insertion of digital technologies in health care [17–19]. To control the spread of COVID-19, most countries have adopted home isolation and quarantine measures [18]. In this scenario, the complications of COVID-19 were more worrying in older patients, which encouraged the implementation of digital health interventions to assist in long-term care and monitoring of chronic diseases at home [20–22].

Monitoring the quality of technology-mediated primary health care at home is relevant, especially at present, when the world is experiencing the end of the COVID-19 global health emergency, the decade of healthy aging, during which the power of digital technologies and health innovation can be harnessed to accelerate the global achievement of health and well-being [20, 21].

HBPC services play a crucial role in the quest to provide high-quality universal health coverage, patient centrality and better quality of life [23]. In this sense, the services of digital health, deployed appropriately, can contribute to the three interrelated pillars of primary health care presented in the *Chronic Care Model* [24] empowered people and engaged communities; multisectoral action for health; and health services that prioritize the delivery of high-quality primary care and essential public health functions—all of which require careful consideration of quality [25].

The impact on the quality of care will be analyzed from the theoretical perspective of Donabedian, which is based on three components: structure, process and result. Structure refers to the resources dimension (such as the availability of digital equipment). The processes (actions) have an organizational dimension (digital health policy guidelines); a technical dimension (applicability and accessibility of digital media, quality and security of shared information, integration of technologies into workflows and use in clinical tasks); and a relational dimension between the care provider and the elderly person (promoting humanized, patient-centered interaction, strengthening the therapeutic bond and empowering the elderly in managing their health). The results are the effects of the use of ICTs in health care, which can be the satisfaction of elderly patients, improved health indicators, reduced complications and improved quality of life [26, 27].

In Donabedian's systemic approach [26, 27], results can be measured by the 7 pillars of quality: effectiveness (ensuring that digital technologies produce desirable results). These include: effectiveness (ensuring that digital care achieves the same results in day-to-day practice); efficiency (use of available resources; optimization with the search for improvements in processes and results); acceptability (adaptation and acceptance of technologies by older people and health professionals); legitimacy of the ethical and legal compliance of digital care; and equity, (equal access and quality in digital health services for all older people, regardless of their individual characteristics).

This scoping study is relevant for its originality in mapping the use and types of digital health interventions and evaluating their impact on the quality of health care for the elderly from Donabedian's theoretical perspective, adapting his framework to evaluate the quality of technology-mediated health care. It has theoretical robustness as it is based on a theory of quality assessment that is widely recognized in the field, methodological rigor, and one of the differential aspects of the methodology is the inclusion of stakeholder consultation as a way of consulting the applicability of the review's results so that they can be accessible to other researchers, managers, caregivers and older people.

Seeking to contribute to improving the quality of primary health care, this review aim to identify and map the uses and types of digital health interventions and their impacts on the quality of home-based primary care for older adults.

## Materials and methods

This is a scoping review that seeks to answer broad questions systematically, with a rigorous, transparent and reliable synthesis of knowledge based on the criteria of the Joanna Briggs Institute (JBI) guided by the theoretical framework for the preparation of scoping reviews [28–30], as well as by the Preferred Reporting Items for Systematic Reviews and Meta-analyses Extension for Scoping Reviews (PRISMA-ScR) [31]. The organization of the extraction, analysis and synthesis of evidence has been updated according to the latest guidance [32]. The choice of this method is anchored in the need for extensive mapping of the literature on this topic, which is emerging. The methodological design containing the nine stages of this study is described in detail in the research protocol already published [33].

### Step 1: Defining and aligning the objective and questions

The following research questions were developed according to the PCC (population—older adults, concept—digital health interventions and context- home-based primary care):

- 1. Which countries use digital health interventions in home-based primary care for older adults?
- 2. What kind of digital health interventions (methods, human resources and technology) are used in home-based primary care for older adults?
- 3. What is the impact of digital health interventions on the quality of home-based primary care for older people?

### Step 2: Developing and aligning the inclusion criteria with the objective and questions

We chose to include publications that addressed the use of digital health interventions in home-based primary care for older adults, were available in full, and responded to the PCC of the research; included primary studies, theoretical communications; and included gray literature, government manuals, reports, as well as dissertations and theses. No time or language filters will be applied to the searches, as the search strategies have been designed to reach a wide range of publications. Duplicate publications (Duplicate publications are those retrieved more than 1 × during the literature search process), literature reviews, editorials, expert opinions, brief communications, and studies with patients under 60 years of age were included as exclusion criteria. This research targets older people, so studies that included patients under the age of 60 were excluded to avoid biasing the results, considering that the health needs and challenges faced by older people are different. Studies that included caregivers and health professionals under the age of 60 were not excluded. 

### Step 3: Describe the planned approach to evidence searching, selection, data extraction, and presentation of the evidence and Step 4: Searching for the evidence

A search strategy adapted to the different bases was used; it was refined by a librarian to improve sensitivity and assertiveness based on the objective of the study, and the standard search strategy is available in Additional file 1.

### Step 5: Selecting the evidence and Step 6: Extracting the evidence

The study selection process was guided by the steps proposed in the Preferred Reporting Items for Systematic Review and Meta-Analyses (PRISMA-ScR). The identified studies were grouped according to the Endnote® reference manager, and duplicates were removed. Rayyan software® was used to assist in blinding the reviewers, who independently performed the double-blind selection. In addition, by title and abstract, conflicts were resolved by a third reviewer. The studies selected by title and abstract were moved to the full-text reading phase. After reading the full texts and validating the final sample, 4 researchers evaluated the compatibility and relevance of the evidence with the objective of the review. The evidence was extracted and organized in an Excel® spreadsheet, and the data were extracted according to the extraction form available in Additional file 2 (for which the form was updated).

### Step 7: Analysis of the evidence and Step 8: Presentation of the results

The results of this scoping review were analyzed as up-to-date suggestions available [32]. Quantitative data were analyzed according to simple descriptive statistics. The basic qualitative content analysis followed the steps proposed by Elo and Kyngäs [34], who described 3 phases of qualitative content analysis for the results of primary qualitative research: I) preparation, ii) organization and iii) reporting. After the preparation and organization of the qualitative data, the data were separated, forming codes and groups of codes according to the reading process and theoretical deepening. According to the research questions, the code groups were aggregated to answer the objective of the study. Those that responded to the impacts of quality were separated and analyzed according to quality dimensions, creating categories of these dimensions, guided by the theoretical framework of Donabedian [26]. The results are presented in tables, figures and tables.

### Step 9: Summary of evidence, conclusions and implications of findings

A summary of the results was preliminarily shared with stakeholders, who were considered to be a mechanism for knowledge transfer and exchange, as well as for developing effective dissemination strategies and ideas for future studies. A summary of the stakeholder comments is available after the discussion. For the development of this stage, the study was approved by the Research Ethics Committee of the authors' institute under CAEE 54853921.0.0000.5292.

## Results

### Inclusion and exclusion criteria

**Fig. 1:**
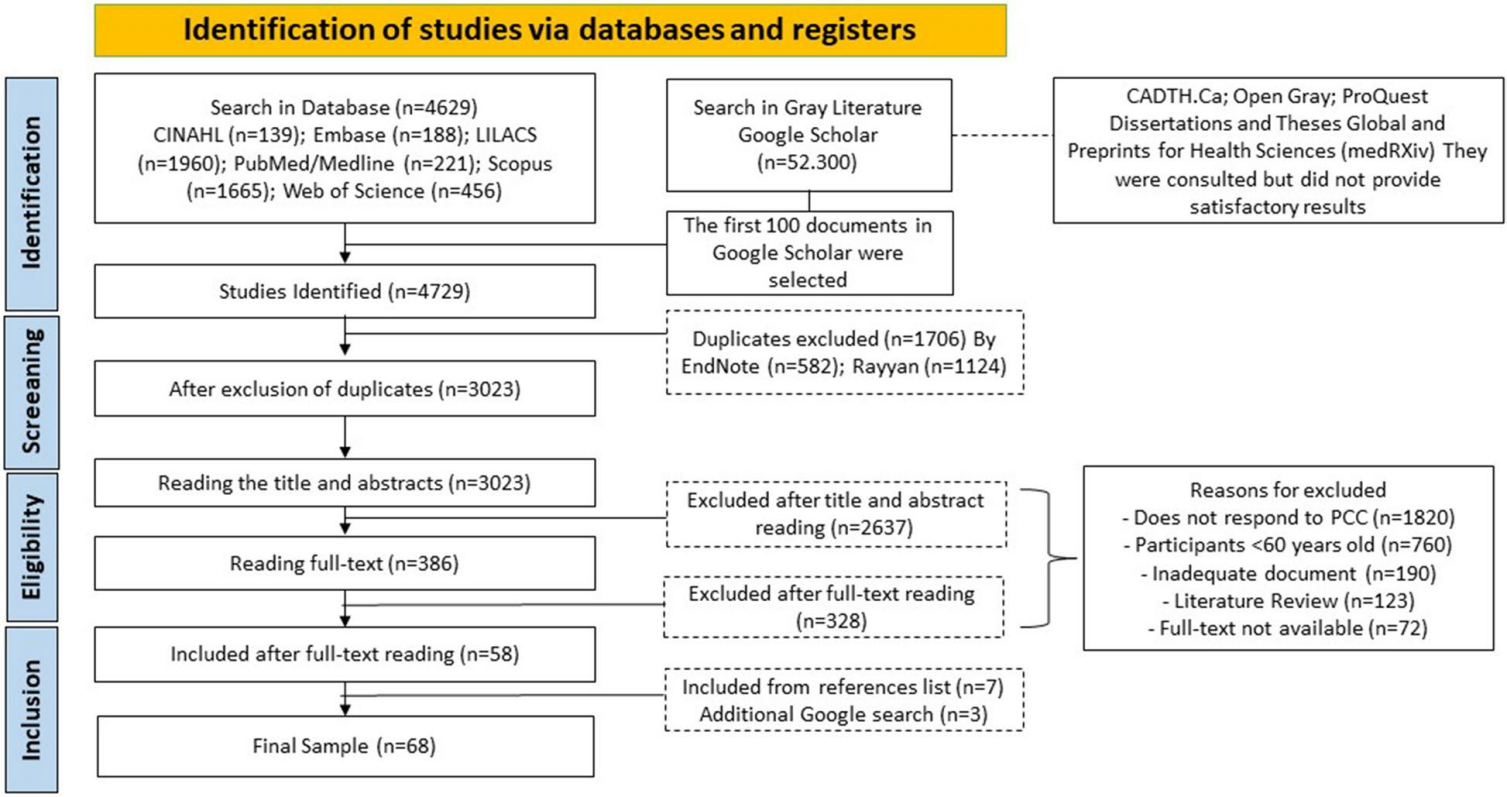
Selection of sources of evidence—PRISMA-ScR

A total of 4,729 documents were identified through the search strategies applied in the databases (LILACS; MEDLINE/PubMed; Scopus; Web of Science; Cinahl and Embase. The gray literature was searched through Google Scholar, Open Gray, “Gray Matters: a practical tool for searching health-related gray literature”, ProQuest Dissertations and Theses Global and Preprints for Health Sciences [medRXiv]). As a result, 68 documents were obtained, 66 of which were scientific articles and 2 of which were reports [35, 36]. The results of the research and the selection process of the studies are summarized in Fig. 1.

### Characteristics of the included studies

**Fig. 2:**
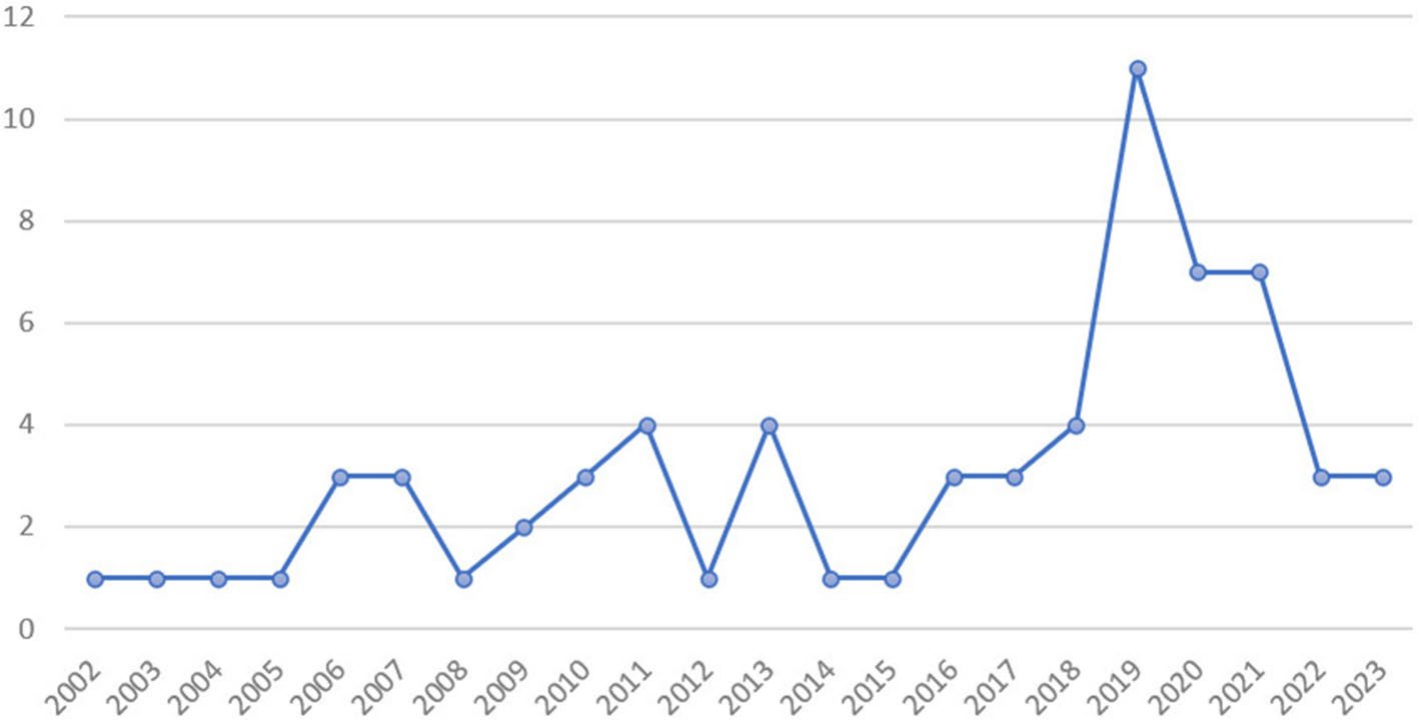
Distribution of the number of publications according to year

The mapping of the sources that make up the results of this study showed that among the 68 (100%) documents, 54.41% (37) are publications from the period of 2002–2018 [35, 37–72] and 45.59% (31) are publications from the last 5 years (2019–2023) [36, 73–102]. Although the highest percentage of evidence was obtained prior to the last 5 years, interest in the topic increased, especially in 2019, when it represented 16.18% of the total sample (as shown in Fig. 2).

In the period corresponding to the years 2020 to 2023, marked by the COVID-19 pandemic, 20 documents were published equivalent to 29.41% of the sample-among which 14 of the evidence was collected or analyzed during the COVID-19 pandemic [36, 74, 75, 83–85, 87, 89, 92, 93, 96, 100–102], thus being influenced by the scenario of the health crisis. In the other 6 documents, data collection took place before the pandemic, and the results were not related to pandemic outcomes [78, 79, 81, 82, 86, 97].

**Fig. 3:**
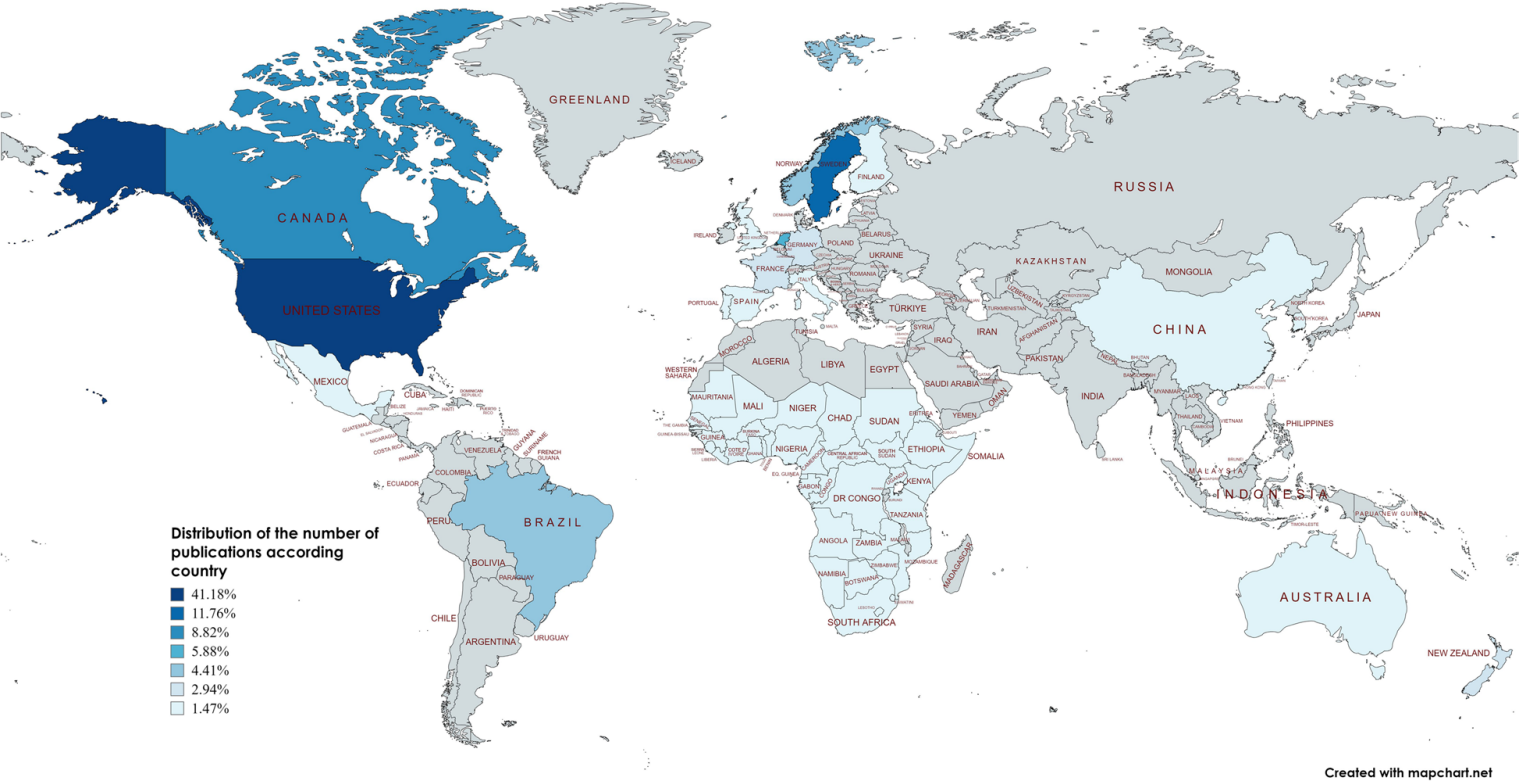
Distribution of the number of publications according to country

It was possible to identify publications about the use of digital health interventions in home-based primary care for older adults from 18 countries and the Sub-Saharan region. Two studies were conducted in 2 countries [55, 91]. The United States [35, 38–43, 46, 47, 49, 50, 52, 53, 55, 58, 61–63, 67, 69, 73, 80, 83, 85, 87, 89, 94, 101] stood out for representing 28 (41.18%) of the studies, followed by Sweden [54, 60, 65, 71, 78, 79, 84, 88] with 8 (11.76%) and Canada [37, 64, 72, 93, 95, 100] which was identified in 6 (8.82%) of the studies, Netherlands [66, 70, 98, 99] with 4 publications (5.88%), Brazil [74, 91, 96] and Norway [77, 81, 90] with 4.41% each. Moreover, data from France [45, 75], Germany [57, 97], and New Zealand [56, 86] were mapped at 2.94% each. The countries with the fewest publications, corresponding to 1 document in each country, were Italy [76], Spain [44], Mexico [48], Portugal [82], Scotland [51], Australia [68], Finland [91], South Korea [55], Hong Kong [92], China [102], England [59] and the sub-Saharan Africa region [36], accounting for 1.47% of the studies individually, as shown in Fig. 3.

The mapping of countries reveals nations with different levels of socioeconomic development. The group of countries considered socioeconomically developed is responsible for the largest number of studies mapped, representing 91.18% of the sample.

### Mapping the terms related to digital health

The publications analyzed revealed the use of the following terms, related to the scope of digital health, “telemonitoring” [39, 40, 43, 49, 53, 59, 65, 67–69, 75, 86, 87, 97–99] was the most used; followed by “telehealth” [38, 47, 51, 59, 61, 63, 80, 83, 89, 93]; “teleassistance” [44, 48, 77]; “telemedicine” [42, 52, 58, 102]; “telerehabilitation” [39, 40, 76]; “teleservice” [37, 94]; “telesurveillance” [72]; “telecare” [64]; “telealarms” [48]; “teleconsultation” [96]; “virtual consultation” [93]; “e-Health” [88]; “teletherapy” [101]; “m-Health” [78]; “health informatics” [41]; “Smart Technology” [102] and “digital health interventions” [102].

### Types of digital health interventions used in home-based primary care for older adults

Regarding the types of digital health interventions used in the HBPC, the results indicate that communication and teleconsultation services were made possible through the use of digital videocall strategies [37, 38, 58, 89, 92, 95]; phone calls [36, 38, 50, 59, 72, 76, 96]; VA app (Video Connect) developed to connect veterans with their healthcare team from anywhere [85]; TV app [82]; WhatsApp [87]; messages [36, 38, 43, 52, 57, 73, 80, 83, 99]; apps (with various functions) [36, 54, 57, 60, 79, 84, 97]; health diary [65]; virtual treatment manual for teletherapy [101]; ZWIP (secure messaging systems complemented by a shared electronic health record) (64), using different types of assistive technologies [36] and devices such as the computer, telephone and television [82, 98]; audio and video device, two-way videophone [43]; tablet [54, 97, 99]; sensors and monitors [41, 45–47, 49, 55, 56, 59, 67–71, 75, 77, 81, 100, 102]. The use of digital platforms for digital health services has also been identified [54, 92, 97, 102].

The home monitoring and sensing of older adults had the greatest prominence in this Scoping Review, and the use of wireless broadband was identified for its success [41, 59]; biosensors [41, 102]; monitoring sensors, activities and behavioral diagnostics [41, 45, 55, 102]; sensors and home devices [86]; environmental monitoring [41]; passive monitoring [46]; tabletop home monitor [47]; health telemonitoring [49, 59]; passive remote monitoring (motion sensors, cameras, drug administration monitoring) [100]; eHAB (telemonitoring system) [68]; Intel Health Guide (telemonitoring) [67, 69]; UAS system (mobility monitoring system, voice response, fire detection, displacement detection and prevention)[70]; Old@Home VHR system (information and communication system) [71]; e-health system [75]; phone and internet-based care ecosystems [59]; telehealth system—Health Buddy Program (HBP) [62]; home monitoring technologies [56]; personal alarms [59, 71]; security alarms, GPS [81, 102].

### Health conditions mapped in digital health-mediated HBPC situations

**Fig. 4:**
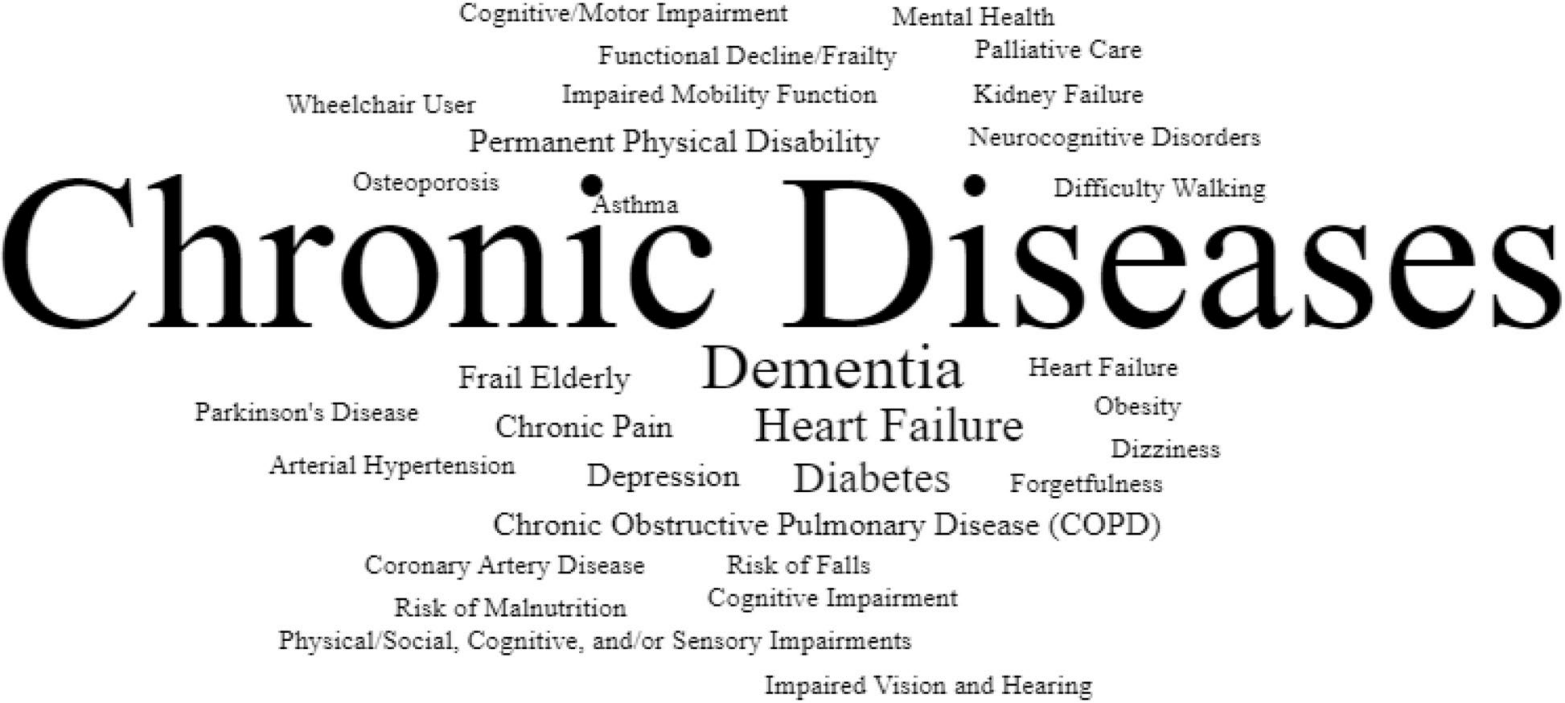
Health conditions addressed by digital means in the HBPC for older people

With the intention of knowing the main health conditions of people who use digital health strategies, Fig. 4 presents a word cloud.

Chronic noncommunicable diseases (NCDs) are the most common health condition [35, 42, 43, 45, 51, 53, 55, 56, 59–61, 63, 64, 67, 68, 70, 75, 76, 83, 85, 89, 96]. Mapping of the impacts on health conditions has shown that digital tools contribute to a substantial improvement in the severity of depression [49]; a reduction in chronic pain, anxiety, sadness and fear (in the face of the epidemiological scenario of the COVID-19 pandemic) [74]; a reduction in complications/falls [76]; early detection of changes in health status [67]; rapid adjustment of treatments; and identification of new clinical conditions [64] and survival [62]. In addition, it has been used as an acceptable and viable resource for improving mental health [101], improving the nutritional status of older adults [98, 99], and improving quality of health and life [42, 52, 64, 72, 82, 93, 102]. In the case of dementia patients, tele-care has been proven to be convenient, comfortable, stress-reducing, time-saving and highly satisfactory [87].

### Impact of the digital HBPC for older people

Considering the theoretical perspective of Donabedian [26, 27], an evaluation of the quality of health care was carried out, and the qualitative data were analyzed; two corpora were formed, namely, positive impacts (Table 1) and challenges and limitations (Table 2). Both were systematized and categorized in the following dimensions of health care quality: organizational, relational/interpersonal, technical and results.

**Table 1:** Positive impacts of digital health interventions on the quality of HBPC for older people

Quality dimensions	Positive impacts of digital health interventions	
**Organizational**	**Access and accessibility**	• It allows care for isolated people, even in remote regions [37, 51, 68, 94, 95] and reduces displacements [38, 58].
		• It can be used to overcome barriers to health access [85].
		• Integration of patient health information with the health service [36, 47, 102].
		• Connecting older people to their informal support networks to assist aging at home [36, 56, 102].
	**Coordination of care**	• Cost reduction [38, 39, 45, 46, 50, 53, 58, 61, 68, 72, 80, 93].
		• Management /Care management [70, 83, 102] of chronic [38] diseases [43, 80].
		• Real-time monitoring [53, 55].
		• Preventive care [102]; contributes to the prevention and promotion of mental and bodily health [74].
		• Improves adherence to treatments and medical recommendations [48].
		• It enabled communication between the older adults and home nurses [54, 60] and thus served as a facilitator in obtaining participatory care.
		• Continuity of care and greater exchange of information to support improved care decisions [73, 102].
**Relational/Interpersonal**	• Reduces caregiver burden [72].	
	• Increased resilience and well-being of both people with NCDs and their home care providers [92].	
	• It improves communication between caregivers, older people and health professionals [66, 71, 86]; improves doctor-patient communication in a pandemic situation [89].	
	• It enables registration and communication between the household and the health service [57].	
**Technique and Results**	• Improving care [75, 88, 90] and access to health care and services [47, 58, 61, 64].	
	• Improved self-care [36, 38, 39, 79]; self-management [39, 78]; autonomy within the home [39, 40, 43]; promotion of functional independence [39]; better understanding of the disease [42], greater participation of older people in their care [67, 84]; better living habits [72]; reduced stress levels [74]; autonomy and independence [97].	
	• It allows you to live longer at home, with a sense of security [97] and well-being [60, 65, 78, 79, 81, 86, 93].	
	• Supports the overall well-being of family and friend caregivers to help frail seniors age at home [100].	
	• Reduction in hospitalization [38, 45, 53, 61–63, 65, 71, 75].	
	• Impacts on health conditions-improved depression outcomes in 70% of patients [49]; reduction of chronic pain, anxiety, sadness and fear (in the face of the epidemiological scenario of the COVID-19 pandemic) [74]; reduction of complications/accidents/falls [76, 102]; early detection of changes in health status [67], allows faster adjustment of treatments and identification of new clinical conditions [64]; improved survival [62].	
	• Acceptable and feasible resource for mental health improvement [101].	
	• Improves the nutritional status of older adults [98].	
	• Better quality of health and/or life [42, 52, 64, 72, 82, 93, 102].	
	• Opportunity for active aging [41].	
	• Technical network that helps maintain the conditions of care provision [44].	
	• Serves more high-risk and high-need patients [73].	
	• Easy usability [54, 65].	
	• Older people felt that technology could be useful in terms of information access and security and were looking for ways to get help with everyday activities [91].	
	• It provides high patient and caregiver satisfaction [63]; satisfaction with service and technology [72, 97]; satisfaction among participants [99].	
	• Continuity of care during the COVID-19 pandemic [96].	
	• The use of telemedicine (tele-service) is convenient, comfortable, stress-reducing, time-saving, and highly satisfying [87].	
	• Means of psychosocial support for the older adults [102]	

Source: Survey data, 2023

**Table 2:** Challenges and limitations in the use of digital health interventions for the quality of health care

Quality dimensions	Challenges and limitations of digital health use in HBPC for older people	
**Organizational**	**Access to technologies and infrastructure**	• Digital divide [78, 85, 89, 93].
		• Lack of analysis of the feasibility of implantation [64, 79].
		• Failures and technical errors in systems [75].
		• Lack of data security assurance [55, 56, 77, 93, 95].
		• Need for data management [55, 62, 95]; information flow [56, 77, 93].
		• Need for technical support (53, 86) and telemedicine support policies [87].
		• Excess information and notifications for family caregivers to manage [100].
		• The technology should be able to process information so that the indicated members of the support network are notified only when the older adults act out of their usual routine [56].
		• Lack of access to appropriate technology (devices and internet) [39, 41].
		• Limited image quality [37].
		• Low usability [42, 56, 73, 80, 86, 93, 99].
		• Absence user-centered design practices, development of eHealth interventions for older adults [56, 99, 102].
		• Interoperability problems [99].
		• Functional problems with the hardware; tablet device with problems [97].
		• Instability in the network, low internet speed [36].
		• Instability in the power grid, the need for reliable internet access [36].
	**People and Team management**	• Lack of training of teams and professionals [37, 76].
		• The need for support from nurses to respond to calls [60, 84].
		• The need for a trained and appropriately sized team [40, 84].
		• Absence of a caregiver at home (it is an obstacle) [89] support of caregivers at home [74, 97, 99].
	**Economic impacts**	• High funding [38, 46, 50, 55, 70, 86] cost [56, 86].
		• Absence of hiring/compensation models [94, 102].
		• Socioeconomic inequalities [78].
		• Need for analysis of logistical elements; infrastructure; refund policy [35].
		• Unknown economic impact [102].
		• The technology should be low-cost [56].
**Relational/ Interpersonal**	• Barriers to acceptance of the use of technologies [35, 41, 43, 60, 62, 63, 67, 73, 84] by older people, caregivers or health professionals.	
	• Poor user confidence [35, 36].	
	• Lack of interest/adherence [66, 82, 85, 98, 102].	
	• Difficulty using technologies—digital literacy [37, 39, 42, 47, 51, 57, 58, 65, 67, 68, 71, 74, 76, 78, 81, 83, 84, 89, 99].	
	• Difficulty in patient communication with the health team [57, 67].	
	• Change in behavior by using the monitoring device [53].	
	• Personal limitations for the use of the tools [42, 60].	
	• Time of use (to learn how to use) [92].	
	• Slowness to obtain response via digital tools [102].	
	• Change in emotions of older people, loss of privacy [102].	
	• Mechanization of health care, problems in humanization—focus on technologies and not on users [102].	
	• It weakens social relationships [102].	
**Technique and Results**	• There was no significant improvement in quality of life [72].	
	• It did not reduce functional decline [69].	
	• It does not replace personal/face-to-face contact [88, 95, 96].	
	• Impact on health care unknown [101].	
	• Assisted living technologies do not help people live with illnesses [59].	
	• Increased anxiety and stress of older adults not being able to manage technologies [60].	
	• Applications should suit the individual treatment path and should not represent an additional burden for the patient or the doctor [97].	
	• Relevance between the choice and the need of patients—Adaptation of technologies to the real needs of each patient [44, 45, 48, 49, 79].	

Source: Survey data, 2023

### Positive impacts

In the case of positive impacts, presented in Table 1, the organizational dimension was reported in two subcategories. The first subcategory addresses the contributions of technologies to facilitating access and accessibility to health care. The second subcategory includes the results focused on the use of digital health technologies, with a focus on care coordination.

The Relational Dimension and Interpersonal dimension portray the use of technologies to improve communication between patients, healthcare professionals, and home care providers. The technical dimension was the most expressive among the positive impacts because 57.97% of the analyzed documents reported some positive outcomes from the use of digital health services covering health care directed at older adults.

### Challenges and limitations

There are challenges and limitations when using digital health interventions for the health care of older adults. Table 2 summarizes how this issue relates to the quality of care. The organizational dimension is organized into three sub-dimensions, namely, access to technologies and to technological infrastructure, challenges related to the management of people and teams and challenges linked to economic impacts.

In the relational/interpersonal dimension, limitations related to the communication of people mediated by technologies, challenges in using digital tools and how this communication affects the relationship between older adults and caregivers are discussed.

In the technical dimension, limitations related to clinical outcomes were found, such as patients reporting no significant improvement in quality of life. The evidence also indicates that digital technologies do not yet have an impact on health care or quality of life.

### Strategies to improve the quality of digital health-mediated HBPCs

**Fig. 5:**
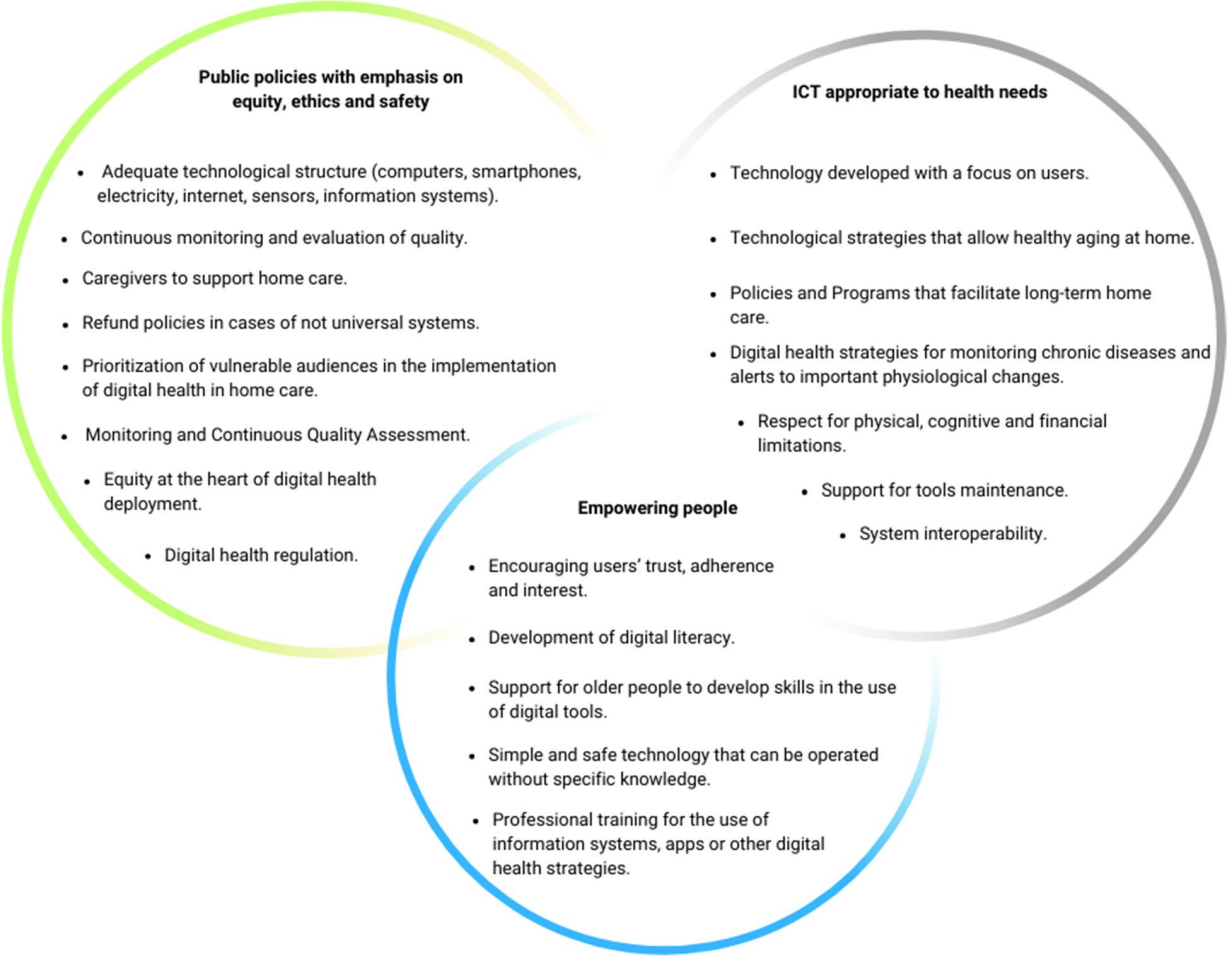
Strategies for improving the quality of care mediated by digital health

To systematize priorities for improving the quality of home care mediated by technologies, we organized a framework to improve the implementation of HBPC, as shown in Fig. 5. Based on the data analyzed, the authors of this review believe that public policies emphasizing equity, ethics and safety should be prioritized to strengthen health systems, people should be trained to use digital interventions in their work process and health care, and ICTs should be developed according to the needs of older people and their caregivers.

## Discussion

The results presented in this study provide a comprehensive overview of the use of digital health interventions in primary home care for older adults. A total of 67 documents were included, published between 2002 and 2023. Developed countries such as the USA, Sweden and Canada have published the most studies on the subject. A wide range of digital interventions were used in home-based primary care for the elderly, including video communication, apps, health monitoring and assistive technologies. The main health conditions addressed were chronic non-communicable diseases, and the digital interventions showed significant improvements in the quality of life and mental health of the elderly. However, challenges were also identified, such as limited access to technologies and communication problems. Several points for discussion emerge from these results, with emphasis on the evaluation of the impacts of digital health on the quality of HBPC for older adults.

The increase in the number of publications on the topic in recent years was identified, which can be justified by the greater incentive of the WHO for the adoption of digital health, which published the first guideline on the topic in 2019 [20]; concomitantly, the implementation of 5G [103], inversion of the age pyramid, in addition to the decade of healthy aging, which encourages PHC as a care ordinator, with emphasis on promoting the quality of life of older people [104], new models of health financing, and the advent of the COVID-19 pandemic [105, 106].

The results indicate that research on the use of digital health services in the HBPC for older people has been conducted on all continents; however, it has concentrated on 2 regions, namely, North America and Europe. These data show the disparity of related research and publications on the use of digital health for the health care of older people in economically different countries. These studies are centered in the U.S., which, although it does not have a universal health care system, but has a model of primary care at home aimed at Veterans (HBPC) and *Medicare* and *Medicaid*. The use of digital health by these models predates the pandemic, and these models are adapted for the home care of older adults [107]. Sweden was the second country most associated with publications and has universal care, including home health and social care for older people, funded mainly by municipal taxes and government subsidies [108].

The mapping of digital tools revealed that, to enable technology-mediated health care, several digital tools can be used. These strategies have improved the accessibility of health care, but they depend on adequate infrastructure. Technological solutions are useful and can be established in developing countries through careful evaluation [109, 110].

In the case of health care for older adults, home and personal monitoring and sensing were highlighted among the types of digital intervention mapped in this Scoping Review. For older people, with cognitive limitations and less ability to handle electronic devices, environmental sensors and wearables based on artificial intelligence technology can be used, such as sensors installed at home, and show benefits in health care [109, 111].

Health technologies are being introduced in domestic environments. Smart homes use Internet of Things (IoTs) technology, Artificial Intelligence, sensors and other equipment to form a technological ecosystem [112, 113]. Digital tools can be used to control and monitor the home environment [112, 114] and thus physiological parameters, such as vital signs, life activities, social interaction, and personal assistance [111, 113], can be monitored.

In this sense, the integrated use of technological devices at home is revolutionizing the field of digital health and health care [114]. Interest in digital solutions is also influenced by the relevance of digital solutions to clinical outcomes and the optimization of resource use [115, 116]. In this way, digital technologies in health can contribute to the Sustainable Development Goals of Health and Well-Being, helping individuals collaborate to achieve universal health coverage and expand access to health services.

Among the people who can benefit from these technologies, as evidenced in this review, are those living with NCDs, as they contribute to improving the prevention and management of health conditions through the use of teleconsultations and the registration and monitoring of patient data, allowing these individuals to play a more active role in health management [22, 117]. Wearables applied to public health have potential for the prevention and control of NCDs in low- and middle-income countries and can be used in the short term to conduct surveys on the risk factors for NCDs, providing important information for building population health profiles [118].

The mapping of the positive impacts of digital health on the HBPC to older people highlights incentives for its adoption and improvements in the quality of care in its organizational, relational/interpersonal,’ technical and results dimensions (with the perspective of Donabedian), while technologies applied to health care can improve the quality of long-term home care [119, 120].

As evidenced in this review, the use of monitoring technology devices is consistent with the specialized literature that reveals the possibility of notable benefits to older people, supporting their independence, mobility, safety and general well-being [121]. In addition to reducing the hospitalization duration, rehospitalization or admission to emergency departments, and health care costs [120, 122], digital health has impacts on the relational dimension with regard to the feasibility of communication, proximity and improvement of human interactions. Thus, this approach can provide updated information in real time and optimize care.

Previous scoping reviews have shown that access and accessibility to health services improved even in the face of social distancing caused by the COVID-19 pandemic through the use of digital health [121, 123, 124]. During the pandemic, older people perceive an improvement in quality of life from the use of technologies related to overcoming the social isolation and loneliness inherent in confinement [125].

Theoretical findings have strengthened the use of digital health, since it can contribute to the coordination of care for older people living at home and strengthen PHC actions [123, 126, 127] by facilitating communication between patients, family caregivers, physicians, and formal outpatient caregivers [127]. The possibility of supporting healthy aging is highlighted while maintaining the autonomy and safety of older people at home [126].

Since the complexity of primary home care requires information organization, technologies should be used to improve the quality of care to ensure that data sets can efficiently serve the entire health process of older people [128].

With regard to the challenges and limitations that interfere with the quality of the HBPC, the evidence warns of the use of ICTs by older people and with a lower level of digital health literacy [129–131]. Inclusion and digital literacy can contribute to adherence to technologies, which are considered key components for solving potential deficiencies; these deficiencies include comfort with digital tools, gender equality in accessing health care, self-managed health conditions and the development of a local workforce specialized in ICTs [132–134]*.*

Access to tools and infrastructure is decisive for the successful implementation of ICTs in health [135]. The digital divide, present in low-and middle-income countries, reinforces the challenges of implementing technological strategies [136, 137]. This phenomenon mainly affects families residing in rural areas, low-income individuals and older people [136]. In this sense, social inequities are responsible for dividing those who can use technologies to improve health care from those who could have their access to health improved by technologies but do not have access to adequate infrastructure.

To democratize the use of digital health, one should invest in adequate technological infrastructure and increase access to broadband Internet and up-to-date computing devices [135]. To achieve egalitarian implementation, sociodemographic criteria must be considered for the technological structuring of territories, seeking to overcome the digital exclusion of economically disadvantaged places and greater integration with PHC [138]. Respecting the specificities of the older population is crucial, as this implies the structuring of HBPCs and support networks so that older people can use technologies in an integrated way with primary care services [139].

The implementation of digital technologies in health care in the context of aging should be guided by ethical precepts [127]. In view of the above, digital surveillance, transparency, data security, collection, storage and use of patient information have attracted increased amounts of attention [140, 141]. It is imperative to invest in an ethical and data privacy framework and legislation to ensure that the data will be adequately protected [133].

It is essential that technologies promote benefits for health care, respect the sociocultural context, be appropriately developed to address health needs, and consider relevance without causing tension or harm, thus contributing to patient satisfaction [142, 143]. In this way, digital health interventions are additional tools for accessing the health professional-patient relationship without ever replacing face-to-face care [144].

According to the literature, despite the growing interest in investing in digital tools in health care, the evidence on the cost-effectiveness of digital health interventions is limited [145]. Thus, it is early to conclude that digital interventions can interfere with the cost-effectiveness of health care.

### Implication of the results

The findings of this study will assist public health policymakers in their decision-making, presenting the difficulties to be faced and the main benefits of digital health. Mapping the digital tools used in the HBPC of older people contributes to choosing the most appropriate digital interventions for each patient, health condition and health care purpose. When making the choice to include digital tools in the home care of older people, managers, health professionals, caregivers and patients should be aware of the strengths and limitations of these strategies, as the implementation of digital health without proper monitoring of the quality of care can increase inequities.

Technology developers must pay attention to the results presented when developing digital interventions that meet the health needs of older people. These should be accessible, intuitive and improve their quality of life, seeking to overcome possible difficulties and limitations, so that the use of technologies does not become a burden in the HBPC of older people.

Further research should be carried out to understand the local reality in different countries, taking into account cultural competence, motivators for professionals to use digital health, and the satisfaction of older people and their caregivers with health technologies. In order to assess the maturity and quality of digital health in HBPC, especially in emerging countries and universal health systems.

### Strengths and limitations in research

The strong point is that this was the first scoping review, with methodological rigor and broad analysis of the evidence, to identify the types of digital intervention used in home-based health care for older people in the context of primary care and to assess the quality of care at the same time. The results allow us to visualize the regions that published their results in the literature to determine the most commonly used tools and the main health conditions of the patients in the context of interest in the study.

As a gap in this research, we can mention the lack of reach of countries that use digital health tools in the home environment for older people but did not publicize their results, preventing them from being identified. This study included the place of interest in the family home; this cut excluded publications that included the place of research community residences, hospitals and long-stay institutions for older adults. A new study focusing on these modalities of residence should be considered.

### Stakeholder consultation

Twenty potential stakeholders were invited via e-mail; among them, eight had confirmed participation and answered questions of interest via Google forms. The participants in this stage were as follows: a doctor working in research related to applications of data science and artificial intelligence in health; a dental surgeon working in the research line of health policy evaluation; a nurse working in a university hospital and member of the University network of Telemedicine REDE RUTE; a lawyer working and researching in the area of digital health; a nurse specialist in gerontology and researcher in public health; a marketing professional and researcher in the area of digital health and infodemic; a researcher in the area of telehealth and its use in the Unified Health System-SUS); Sanitarian and public health manager.

The stakeholder consultation strengthened the discussion, pointing out the most relevant results and weaknesses in the presentation of these results that were corrected at the preparation stage of the article. Regarding strategies for the dissemination and sharing of results, stakeholders suggested scientific socialization in national/international journals. The technical sharing of the results (science translation) among managers with decision-making power was suggested to enable the implementation, monitoring and evaluation of interventions in digital health.

The results of this consultation indicate that the authors should be concerned with promoting the participation of professionals, managers and other stakeholders in the formulation and implementation of guidelines and policies for the implementation of digital health to reduce health inequities. In addition, to reach the population, articulating with figures of political influence and social representatives is relevant. Stakeholders encouraged dissemination at health events in the form of banners, open exhibitions, short videos and executive summaries, and sharing on social networks and official websites of the institutions to which the researchers are linked. It was suggested that the theme of this research be expanded in the training spaces of future health professionals.

## Conclusion

The present scoping review mapped and identified the uses and types of digital health interventions and their impacts on the quality of primary home care for older adults. The findings presented here will be useful for researchers, health professionals, technology developers, managers and users to understand the current landscape and the actions needed to improve technology-mediated health care. In addition, the results point to the relevance of the use of digital health and the types of technologies most commonly used in clinical situations that can be mediated by digital health.

Digital health is playing an increasingly important role in improving the quality of home-based health care for older people through the use of a multidisciplinary approach. However, to maximize its potential, it is crucial to address the challenges identified and ensure that interventions are tailored to the individual needs of patients, as well as to investment in digital literacy, age-appropriate technologies and policies for equitable deployment of digital health. This approach can help promote healthy aging, improve the management of NCDs, and provide high-quality care to older adults worldwide.

Based on the results presented, it is possible to establish priority themes that should be addressed and researched, such as the challenges for equitable implementation of digital health in emerging countries, the sustainability of universal health systems and home care, policies for assessing the quality and safety of digital health technologies used in the HBPC, new business models of digital services and the financing of digital health in the context of PHC. Advances in the health area must be distributed according to the concept of equity, overcoming socioeconomic inequities.

### Supplementary Information

Supplementary Material 1.

Supplementary Material 2.

## Data Availability

Data is provided within the manuscript or supplementary information files.

## Abbreviations

HBPC: Home Based Primary Care
IoTs: Internet of Things
JBI: Joanna Briggs Institute
NCDs: Chronic noncommunicable diseases
PHC: Primary Health Care
PRISMA-ScR: Preferred Reporting Items for Systematic Reviews and Meta-analyses Extension for Scoping Reviews
