# Correction: Digital home care interventions and quality of primary care for older adults: a scoping review

**DOI:** 10.1186/s12877-024-05280-y

**Published:** 2024-08-08

**Authors:** Ísis de Siqueira Silva, Aguinaldo José de Araújo, Rayssa Horácio Lopes, Cícera Renata Diniz Vieira Silva, Pedro Bezerra Xavier, Renan Cabral de Figueirêdo, Ewerton William Gomes Brito, Luís Velez Lapão, Cláudia Santos Martiniano, Vilani Medeiros de Araújo Nunes, Severina Alice da Costa Uchôa

**Affiliations:** 1 Postgraduate in Collective Health, Federal University of Rio Grande Do Norte, Natal, Brazil; 2 Health School, Federal University of Rio Grande Do Norte, Natal, Brazil; 3 Technical School of Health of Cajazeiras, Teacher Training Center, Universidade Federal of Campina Grande, Cajazeiras, Brazil; 4 Postgraduate in Health Sciences, Federal University of Rio Grande Do Norte, Natal, Brazil; 5 Postgraduate in Family Health, Federal University of Rio Grande Do Norte, Natal, Brazil; 6 Public Health Department, Federal University of Rio Grande Do Norte, Natal, Brazil; 7 WHO Collaborating Center on Health Workforce Policy and Planning, IHMT, Universidade Nova de Lisboa, Lisbon, Portugal; 8 UNIDEMI, Department of Mechanical and Industrial Engineering, Nova School of Science and Technology, Caparica, Portugal; 9 Laboratório Associado de Sistemas Inteligentes, Escola de Engenharia Universidade do Minho, Campus Azurém, Guimarães, 4800-058 Portugal; 10 Department of Nursing, State University of Paraíba, Campina Grande, Brazil

**Correction: BMC Geriatr 24, 507 (2024)**

10.1186/s12877-024-05120-z

Following publication of the original article [1], the authors reported that the affiliations of author Luís Velez Lapão was incorrect. The correct affiliations are presented correctly in this correction article.

The original article [1] has been corrected.
