# Supplementary material for: Digital home care interventions and quality of primary care for older adults: a scoping review
Source: BMC Geriatr. 2024 Jun 10;24:507. doi: 10.1186/s12877-024-05120-z (PMC11163791; doi:10.1186/s12877-024-05120-z)
Supplement: Supplementary file 1 — Supplementary Material 1. [file 12877_2024_5120_MOESM1_ESM.pdf]

**Additional file 1.** Search Strategy*

| | |
|---|---|
| **Population** | aged OR elderly OR "aged patient" OR "aged people" OR "aged person" OR "aged subject" OR "elderly patient" OR "elderly people" OR "elderly person" OR "elderly subject" OR "senior citizen" OR senium OR "middle aged" OR "middle age" OR "old person" OR "old people" OR "very elderly" OR centenarian OR centenarians OR nonagenarian OR nonagenarians OR octogenarian OR octogenarians OR "very old" OR "oldest old" OR "aged 60" OR "aged 70" OR "aged 80" OR "aged 90" OR "aged 100" OR "60 years" OR "70 years" OR "80 years" OR "90 years" OR "100 years" |
| **Concept** | Telemedicine OR "m-Health" OR "Mobile Health" OR "mobile healthcare" OR mHealth OR eHealth OR "tele medicine" OR teleconsultation OR "long distance consultation" OR "tele-consultation" OR "telephone consultation" OR "electronic consultation" OR "e-consultation" OR econsultation OR "remote consultation" OR teleconsultations OR telehealth OR "e-health" OR "tele-health" OR telediagnosis OR "remote diagnoses" OR "remote diagnosis" OR "remote diagnostics" OR "telediagnosis" OR telediagnoses OR telediagnostics OR telemonitoring OR "distant monitoring" OR "distant patient monitoring" OR "remote monitoring" OR "remote patient monitoring" OR "tele monitoring" OR "video consultation" OR "telemedicine video-consultation" OR videoconsultation OR telepharmacy OR "tele-pharmacy" OR telenursing OR "tele-nursing" OR "digital health" OR "digital-health" |
| **Context** | "home care services" OR "home care service" OR "domiciliary care" OR "home health care" OR "home care" OR "domestic health care" OR "domestic healthcare" OR "domiciliary health care" OR "domiciliary healthcare" OR "home care agencies" OR "home care program" OR "home care programme" OR "home care service" OR "home health nursing" OR "home health care nursing" OR "home healthcare" OR "home help" OR "home nursing" OR "home service" OR "home treatment" OR homecare OR "homemaker service" OR "homemaker services" |

* The standard strategy has been adapted as necessary for use in the databases.
