# Supplementary material for: Digital home care interventions and quality of primary care for older adults: a scoping review
Source: BMC Geriatr. 2024 Jun 10;24:507. doi: 10.1186/s12877-024-05120-z (PMC11163791; doi:10.1186/s12877-024-05120-z)
Supplement: Supplementary file 2 — Supplementary Material 2. [file 12877_2024_5120_MOESM2_ESM.pdf]

Additional file 2. Standard data collection instrument

**Study characteristics**	
Study title	
Main author	
Journal	
Year of publication	
Study country	
Language	
Study design/ type of file	
Study population	
Study objective	
Research question	
Participants	
Main results	
**Research question data**	
Type of digital health strategy used	
Purpose of use	
Assess the impact of using digital health strategies on the quality of home-based primary care	
Agent responsible for care actions	

Availability of digital health strategies by PHC	
Older adult's health condition	
Age of participants	
Potential/opportunity Limitation or challenge	
